# Cortical pain induced by optogenetic cortical spreading depression: from whole brain activity mapping

**DOI:** 10.1186/s13041-022-00985-w

**Published:** 2022-12-05

**Authors:** Chenghui Pi, Wenjing Tang, Zhishuai Li, Yang Liu, Qi Jing, Wei Dai, Tao Wang, Chunxiao Yang, Shengyuan Yu

**Affiliations:** 1 College of Medicine, Nankai University, Tianjin, China; 2 Department of Neurology, The First Medical Centre, Chinese PLA General Hospital, Beijing, China; 3 The State Key Laboratory for Management and Control of Complex Systems, Institute of Automation, Chinese Academy of Sciences, Beijing, China; 4 School of Life Sciences, University of Science and Technology of China, Hefei, China

**Keywords:** Cortical spreading depression, Whole-brain activity mapping, Cortical pain

## Abstract

**Background:**

Cortical spreading depression (CSD) is an electrophysiological event underlying migraine aura. Traditional CSD models are invasive and often cause injuries. The aim of the study was to establish a minimally invasive optogenetic CSD model and identify the active networks after CSD using whole-brain activity mapping.

**Methods:**

CSD was induced in mice by light illumination, and their periorbital thresholds and behaviours in the open field, elevated plus-maze and light-aversion were recorded. Using c-fos, we mapped the brain activity after CSD. The whole brain was imaged, reconstructed and analyzed using the Volumetric Imaging with Synchronized on-the-fly-scan and Readout technique. To ensure the accuracy of the results, the immunofluorescence staining method was used to verify the imaging results.

**Results:**

The optogenetic CSD model showed significantly decreased periorbital thresholds, increased facial grooming and freezing behaviours and prominent light-aversion behaviours. Brain activity mapping revealed that the somatosensory, primary sensory, olfactory, basal ganglia and default mode networks were activated. However, the thalamus and trigeminal nucleus caudalis were not activated.

**Conclusions:**

Optogenetic CSD model could mimic the behaviours of headache and photophobia. Moreover, the optogenetic CSD could activate multiple sensory cortical regions without the thalamus or trigeminal nucleus caudalis to induce cortical pain.

## Highlights

1. We established an optogenetic mouse CSD model without irritation dura, which could mimic the behaviors of headache and photophobia.
2. It was the first time to depict a broad, unbiased neuronal activity map throughout the mouse brain after CSD.
3. Results of whole brain image and immunofluorescence staining showed that optogenetic CSD could activate multiple sensory-related networks without activating thalamus or TNC to induce cortical pain.

## Background

Cortical spreading depression (CSD), a propagated wave of depolarization of neurons, glia and vessels followed by suppression of brain activity, has been presumed as a physiological substrate of migraine aura [1]. Previous CSD studies have suggested that pin prick and potassium chloride (KCl) stimulation of the visual cortex activates the nociceptive afferents of the meninges to increase the long-term firing of trigeminal nucleus caudalis (TNC) neurons [2, 3]; however, KCl-induced CSD could also directly regulate the firing of TNC neurons through subcortical pathways [4, 5]. Notably, previous studies have used invasive and potentially injurious methods to induce CSD, such as placing high-concentration KCl cotton on meninges or pin prick into the cortex, thereby casting doubt on whether the pain responses were the result of CSD or direct stimulation of the dura mater. As a result, the pathway by which CSD induces the headache attack remains controversial [1, 6, 7]. The noninvasive optogenetic CSD model using Thy-1/ChR2-YFP mice could induce CSD repeatedly without causing tissue damage, however the ChR2 expression was not enough in occipital cortex [8, 9]. Masvidal-Codina et al. established a new optogenetic CSD model using hSyn-ChR2-EYFP virus, which makes it possible to induce CSD in any cortical region [10].

Glutamate might play a critical role in CSD generation, as glutamate receptor activators and inhibitors can induce and inhibit the spread of CSD, respectively [11, 12]. Familial hemiplegic migraine type 1 causes increased susceptibility to CSD by increasing glutamate release [13]. Therefore, we induced CSD by stimulating glutamatergic neurons without irritating the dura mater.

Obtaining a map of neuronal activity map is important for understanding the dynamic changes in neural networks. Immediate early genes (IEGs), such as c-fos, are linked to recent neuronal activity [14]. Some studies have confirmed that the expression of c-fos is upregulated in the anterior cingulate cortex, amygdala, striatal and hippocampus [15, 16]. However, the researchers only selected the region of interest and no previous studies have depicted a broad, unbiased IEGs activity map throughout the brain. Volumetric Imaging with Synchronized on-the-fly-scan and Readout (VISoR) can quickly and accurately obtain the whole brain cell activity at different levels of details [17, 18]. Using VISoR and optogenetic techniques, we aimed to establish a new model of CSD and described patterns of whole brain activity after CSD.

## Methods

### Animals

Male calcium/calmodulin-dependent protein kinase II alpha (CamkIIa) Cre-recombinase mice (CamkIIa-Cre) of 8–12 weeks were used in this study. It was reported that the threshold for CSD is significantly lower during diestrus in female mice [19]. On the contrary, the threshold of CSD was steady in male mice. Therefore, only male mice were used in the CSD research to exclude the influence of diestrus [20, 21]. Mice were maintained with group housing (n = 3–5 per cage) on a 12 h light–dark cycle (light was on from 9:00 a.m. to 9:00 p.m.) at 23–24 °C and 55 ± 5% humidity with food and water ad libitum. The mice were randomly assigned to CSD or control groups with the rand function of Excel software. All experimental protocols for this study were approved by the Chinese People Liberation Army General Hospital Animal Experimentation Committee and conducted in accordance with the National Institutes of Health Guide for the Care and Use of Laboratory Animals.

### Virus injection

CaMKIIa-Cre mice (8–12 weeks old) were anaesthetized with isoflurane (induction 4%, maintenance 1.5%) and placed in a stereotaxic frame (69101, RWD Life Science, Shenzhen, China). During the operation, the body temperatures of anaesthetized mice were kept constant at 37 ± 0.5 °C using a heating pad and protective ophthalmic gel was applied to avoid eyes dryness. The skull was exposed under antiseptic conditions and a small burr holes were stereotactically made with a thin drill over the right visual cortex (3.5 mm posterior to the Bregma, 2 mm lateral to the midline). 400 nl rAAV-EF1α-DIO-hChR2(H134R)-EYFP-WPRE-hGH (AAV2/9, 4.07 × 10^12^ genomic copies per mL, PT-0001) was injected in the right visual cortex using a glass micropipette (tip diameter ~ 15 µm) attached to microsyringe (5 μL, Hamilton), which were connected to a syringe pump (TJ-1A, Longerpump, China). The 400 nl rAAV-EF1α-DIO-EYFP-WPRE-hGH (AAV2/9, 5.24 × 10^12^ genomic copies per mL, PT-0012) was injected into the right visual cortex in the control group. Over 10 min period, 400 nl of the virus was injected at a depth of 0.3 mm from the surface of the cortex. The micropipette was remained for 10 min at the end of infusion to allow for virus diffusion. Fibre optics were implanted at least 4 weeks after virus injection.

### Optogenetic induction of CSD

Mice were placed in a stereotaxic frame on a homoeothermic heating pad with continuous monitoring of the core temperature (37 ± 0.5 °C). An incision was made to expose the skull surface of each mouse head. The right hole in the visual cortex remained after the virus injection. For photostimulation, a 400-μm diameter fibre optic cannula (2.5 mm O.D., 0.37NA; Newdoon, Hangzhou) was placed on the visual burr hole and connected via a flexible optic fibre to a 465 nm LED light source (2009, Plexon, USA) controlled by an optogenetic controller (184000NQV, Plexon, USA). The stimulation was 10 s at 1 mW increments (maximum of 10mW) every 10 min until a CSD was detected (defined as a spreading DC-potential shift of at least 5 mV amplitude and a spreading reduction in cerebral blood flow of at least 20% relative to baseline [9]). The output power at the tip of optic cannula was calibrated using a photometer (PM100D Thorlabs, Dachau Germany). Non-contact laser flowmetry (PeriCam PSI HR, PrimedAB, Sweden) was placed above the mouse skull to detect changes in microcirculatory blood flow perfusion and a tungsten electrode was placed in the frontal cortex (1 mm anterior to Bregma, 2 mm lateral to the midline, 0.3 mm depth to Bregma) to detect DC-potential. The signals were filtered with DC-400 Hz low-pass filter, 30 × amplified and digitized at 1000 Hz sampling rate (NeuroScan SymAmp system, Australia). The waveform was then processed by DC-0.1 Hz to filter the high-frequency noise using Matlab (Mathworks, versionR2022a, Natick, MA, USA).

The cortical fluorescence distribution of each mice in the experiment was confirmed by confocal microscope (TCS SP8, Leica, Germany). If the fluorescence distribution was incorrect or the volume of fluorescence distribution is less than 1 mm^3^ [10], the mouse would be excluded.

### The whole-brain c-fos imaging

Once the range of thresholds was determined, the CSD was induced in new mice to image the whole brain. If the laser flowmetry detected the typical blood flow perfusion of CSD, the CSD model was considered successfully established. The mice were sacrificed at 3 h after the CSD induction.

For brain harvesting, the mice were anesthetized by intraperitoneal injection of 1.25% avertin (20 mg/kg). They were then transcardially perfused with 40 mL phosphate-buffered saline (PBS), followed by 20 mL of 4% paraformaldehyde (pH 7.4). Dissected brains were post-fixed in 4% paraformaldehyde for 24 h at 4 °C.

The fixed brains were cut into 300 um slices by vibrosclicer (Compresstome VF-300 Precisionary Instruments). Thereafter, the slices were transferred to a clearing solution for 12 ~ 24 h at 37 °C and gently oscillated. After clearing, the sliced samples were washed with 0.1% PBST for 3 times. For immunofluorescence staining, the sections were loaded into 12 well plates, blocked with blocking solution (5% bovine serum albumin, 0.3% TritonX-100 PBS) for 1 h, and then incubated with primary antibody (rabbit anti-c-fos, CST 9F6, diluted 1:1500) at room temperature overnight, and then washed with 0.3% PBST for 3 times. The secondary antibody (AF488 labeled donkey anti rabbit IgG, Jackson Immuno Research Labs 711–545-152, dilution 1:500) was incubated at room temperature for 5 h, and then washed with 0.3% PBST. The samples on the imaging slide were incubated in the 4% HMS for 4 h, and then transferred to the imaging chamber filled with the refractive index matching solution overnight. Synchronous beam-scan illumination and camera frame readout were used to obtain 16-bit images and the sample stage moved linearly in the X-direction. The size of the voxel was 0.5 × 0.5 × 3.5 μm^3^.

Image reconstruction was performed using run_VISoR (version 0.7.1) to generate 4 μm 16-bit images. To match with the brain atlas, ImageJ software (version1.8.0) was used to merge 4 μm images into 25 μm images. For cell detection and counting, 4 pieces of 25 μm brain slices were selected to Ilastic software (version1.3.3 post 3) to adjust the threshold values of background and c-fos positive cells. Thereafter, a single c-fos positive cell was segmented and marked. Smooth processing was used to reduce noise, and the threshold values were selected according to the situation of the marking points. After machine learning training, the brain slices of the whole sample were processed in batches to generate a table containing c-fos positive cells coordinate information. Run_VISoR was used to generate files that automatically matched brain maps. The brain slices were then opened with Freesia software (version2.0.3) to adjust the brain slices according to the size and position of the standard Allen Brain Atlas. Finally, the file containing c-fos positive cells were inputted into Freesia. Furthermore, the number and density of c-fos positive cells in each brain area was output.

### Immunofluorescence staining

The process of the KCl- induced CSD model was similar to the optogenetic CSD model. A craniotomy was performed at the visual cortex (3.5 mm posterior to the Bregma, 2 mm lateral to the midline) and 2 µL KCl (1 mol/L) was applied to the dura to induce CSD. 2 µL NaCl (0.9%) was applied to the dura in the control group. The mice were sacrificed 3 h after the KCl or NaCl applied. The optogenetic CSD model and control group (EYFP group) were established as previous described. Then the brains were post-fixed with 4% PFA for 24 h and cryoprotected in 15% and 30% sucrose solution in turns. The fixed brains were cut into 30-µm thick slices for immunostaining. The ventral posteromedial thalamic nucleus (VPM) (Bregma, − 1.22 mm to − 2.46 mm), ventral posterolateral thalamic nucleus (VPL) (Bregma, − 0.94 mm to − 2.18 mm) and reticular thalamic nucleus (Rt) (Bregma, − 0.46 mm to − 1.94 mm) in the thalamus and I and II laminae of TNC (obex, − 0.5 mm to − 3 mm) were acquired. Free-floating sections were blocked with phosphate-buffered saline (PBS) containing 10% goat serum and 0.25% TritonX-100 for 2 h and incubated with primary antibodies(rabbit anti-c-fos, CST 9F6, diluted 1:1500) at 4 °C overnight. Sections were washed 3 times with 0.25% PBST and then incubated with secondary antibodies(goat anti-rabbit 488, ab150077, diluted 1:1000) at room temperature for 2 h. Lastly, the sections were washed with 0.25% PBST for three times. The slides were then scanned by confocal microscope (TCS SP8, Leica, German).

### Measurement of periorbital thresholds

For behavioral experiment, a fiber optic ferrule was placed above the visual cortex (3.5 mm posterior to the Bregma, 2 mm lateral to the midline) and fixed on the skull with skull screws, cyanoacrylate glue and dental cement after at least 4 weeks of virus injection. If the laser flowmetry detected the typical blood flow perfusion of CSD and the volume of fluorescence distribution is more than 1 mm^3^, the mouse would be included in the behavior analysis. The mice were anesthesia with isoflurane to induced CSD before behavioural test in order to be consistent with previous experimental conditions.

The periorbital thresholds were measured 1 week after ferrules implantation. The test were conducted from 10:00 to 16:00. In addition, the mice were acclimated to the testing environment for 3 days before baseline testing and the mice were acclimated to the testing environment for 1 h before testing. Mechanical thresholds were tested by an evaluator blinded to group allocation with the “up and down” method. The von Frey monofilaments were applied perpendicular to the surface superior-medial to the right and left eyes with a force that allowed the monofilament to bend 3 s. The positive responses included head withdrawal, vigorous turning and screeching. A monofilament was applied 5 times and the minimum bending force able to 3 occurrence of positive responses was expressed as “X”. Periorbital thresholds were determined at 0.5 h, 1 h, 2 h, 3 h and 24 h after CSD.

### Open field test

The apparatus was 30*30*40 cm box in a quiet room illuminated to 30 lx. The mice were brought into the test room 3 h in advance to adapt to the environment. The mice were placed in the center of the box for 30 min immediately after the CSD stimulation. The central area travel distance, grooming, head shaking and freezing of mice were recorded. The field was cleaned with 75% ethanol between tests. The recording software was CinePlexStudioV3 (Plexon, USA), and the behavioral analysis software was CinePlexEditorV3 (Plexon, USA). Freezing behavior was recognized when mice suddenly became immobile except for breathing. Freezing episodes could interrupt any on-going activity such as walking, rearing, grooming or eating. The four paws are on the ground and the eyes are dull, may be accompanied by autonomic symptoms, arched back, piloerection, defecation, urination, proptosis and ear retraction when freezing occurs [22]. Facial grooming: forepaws and hindpaws move on the head or face. Body grooming: forepaws, hindpaws, tongue or incisors touch the body. Head shake: a rhythmic, rapid head shake.

### Elevated plus-maze test

The elevated plus-maze consisted of two open arms (30*6 cm), two closed arms (30*6*20 cm) and a central platform (6*6 cm). The elevated cross is about 40 cm high from the ground. Each mouse was placed onto the central area, heading towards the same open arm. The mice were brought into the test room 3 h in advance to adapt to the environment. Each mouse was videotaped for 20 min immediately after the CSD stimulation. Time spent, travel distance and number of entries into open arms was analyzed using the supermaze software (Shanghai Xinruan, China). The field was cleaned with 75% ethanol between test.

### Light aversion and motility assays

Photophobia was measured immediately after light stimulation, and each mouse was measured for 20 min. The mice were brought into the test room 3 h in advance to adapt to the environment. The light and dark box size was 30 * 30 * 30 cm. The box was partitioned equally. There was a 7 × 7 cm hole in the middle of the partition, which allowed the mice to move freely. The light box was composed of 6 white organic plates, the light intensity at the center of the open box was 3600 lx. The dark box was composed of 6 black organic plates with a light intensity of 60 lx in the center, and 15 lx in the four corners. Infrared cameras were installed over the cover of light box and dark box. The surface of the partition in light and dark box was white and black, respectively. Twenty built-in LED light sources were placed on the top of the light box, and the power of each light source was 1 W. The light and dark box temperature was 27 ± 0.5 °C and 26 ± 0.5 °C, respectively. The temperature was within the appropriate temperature range for mice (26–29 °C), which was unlikely to make mice have location preference due to temperature. The light/dark mobility data were collected by SuperMaze Vesion3.3 (XinRuan, Shanghai, China). In the light and dark boxes, the total ambulatory distance and resting time were recorded.

### Statistical analysis

SPSS (version 26) was used for statistical comparisons. Data were analyzed between the treatment groups (CSD vs control). One-way repeated measures ANOVA with Bonferroni post hoc test was used for the periorbital threshold and light aversion behaviours. Student’s t-test was used for c-fos analysis. Normality and equal variance tests were performed for all analyses. A *p-*value of 0.05 or less was considered as statistical significant.

## Results

### Optogenetic stimulation-induced CSD

**Fig. 1:**
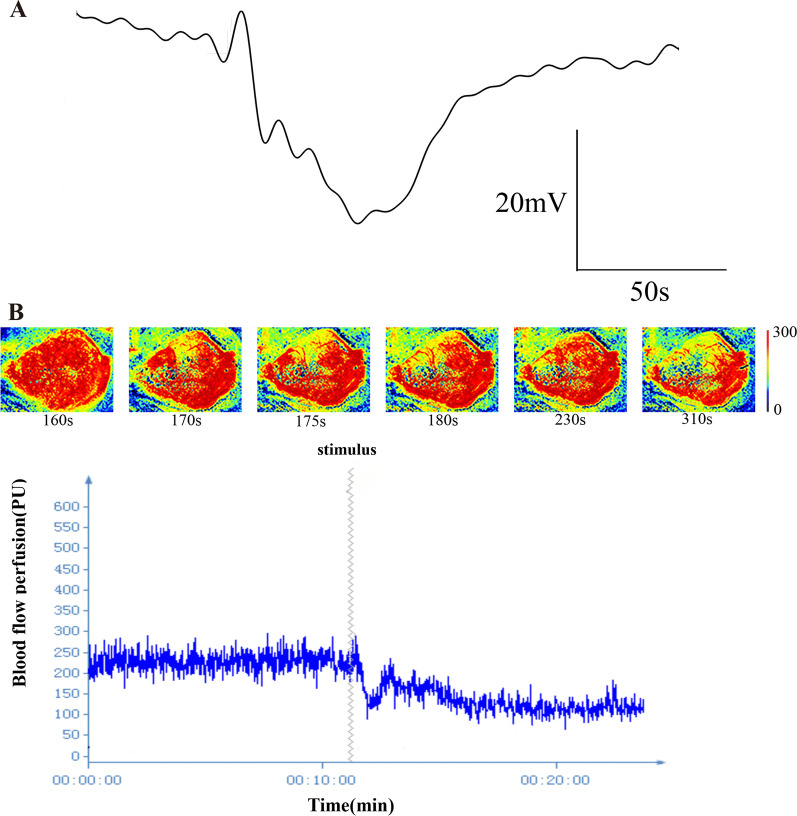
CSD waveform and cerebral blood flow changes. **A** Electrophysiological recordings showed a broad negative wave were recorded in motor cortex, which was consistent with the characteristics of CSD waveform. **B** Representative blood flow perfusion from the ipsilateral cortex, consisting of the initial hypoperfusion followed by a transient recovery of perfusion and a long-lasting oligemia. The zigzag line represented the light stimulation

CSD was successfully induced in the visual cortex using 4–8 mW 465 nm light illumination as evidenced by the characteristic propagating DC-potential shift recorded in the motor cortex and typical blood flow changes. CSD (n = 10) amplitude (20.3 ± 2.5 mV), duration (72.7 ± 18.12 s) and propagation rate (5.87 ± 0.72 mm/min) were consistent with those induced by other methods [9, 23] (Fig. 1A, B). Electrophysiological recordings showed a broad negative wave recorded in the motor cortex, which was consistent with the characteristic of the CSD wave. The typical blood flow perfusion changes, including initial hypoperfusion, transient hyperemia and the long-lasting oligemia, were recorded by the laser flowmetry.

### Behavioural results

**Fig. 2:**
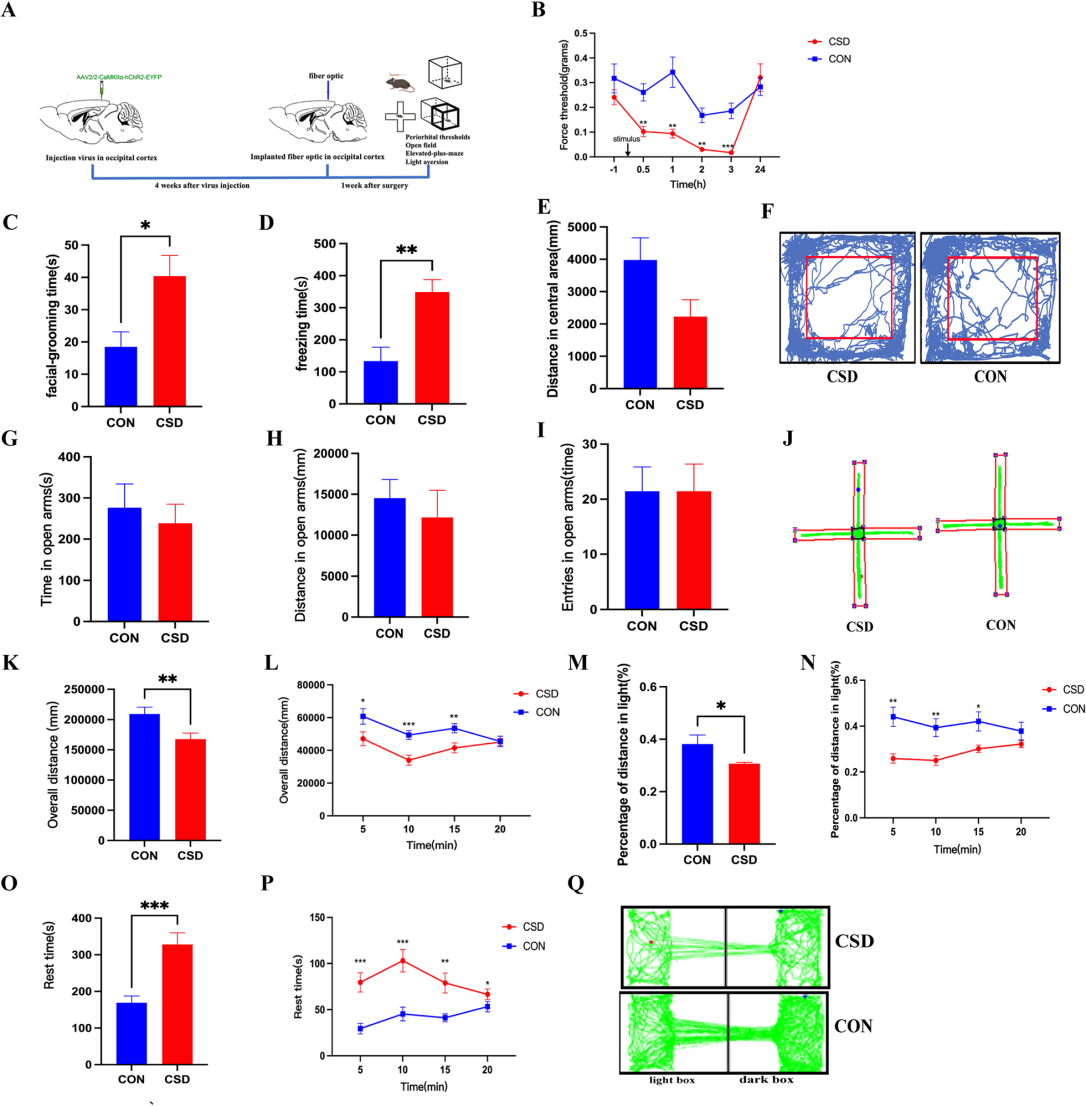
CSD produce periorbital mechanical allodynia, increased facial grooming, freezing and light-aversion behaviors. For all panels, the mean ± SEM is shown, *p < 0.05, **p < 0.01, ***p < 0.001. **A** Schematic diagrams of experiments and behavior tests. **B** Periorbital mechanical thresholds in the CSD group (n = 14) showed a significant reduction at 0.5 h, 1 h, 2 h and 3 h, gradually decreasing to a valley at 3 h and returning to baseline at 24 h compared with the control group (n = 14) (*p* = 0.0043, *p* = 0.0088, *p* = 0.0027, *p* = 0.0008 respectively, one-way repeated ANOVA). **C** Compared with the control group (n = 8), the time of facial grooming of the CSD group (n = 8) were significantly longer (*p* = 0.0228, t-test). **D** Compared with the control group (n = 8), the time of freezing behaviors of the CSD group (n = 8) were significantly longer (*p* = 0.0024, t-test). **E** There was no significant difference in travel distance in the central area between the two groups. **F** Schematic traces of open field tests. **G**–**I** There was no difference in the time spent, travel distance and entries of open arms between the CSD group (n = 8) and the control group (n = 6). **J** Schematic traces of elevated plus-maze tests. **K** The overall distance of CSD group (n = 31) was significantly less than that of control group (n = 27) (*p* < 0.001, t-test). **L** The mice in CSD group travelled less than those in control group (*p* = 0.036, *p* = 0.001, and *p* < 0.001 respectively, one-way repeated ANOVA). **M** The percentage distance in light box of CSD group (n = 31) was significantly less than that of control group (n = 27) (*p* = 0.0246, t-test). **N** The mice in CSD group traveled less in light box at 5, 10 and 15 min interval (*p* = 0.015, *p* = 0.0099, and *p* = 0.048 respectively, one-way repeated ANOVA). **O** The rest time in dark box of CSD group (n = 31) was significantly more than that of control group (n = 27) (*p* < 0.001, t-test). **P** CSD group spent significantly more time resting in the dark box compared with control group (*p* < 0.001, *p* < 0.001, *p* = 0.003 and *p* = 0.025 respectively, one-way repeated ANOVA). **Q** Schematic traces of light-aversion tests

Periorbital mechanical thresholds in the CSD group (n = 14) showed a significant reduction at 0.5 h, 1 h, 2 h and 3 h, gradually decreasing to a valley at 3 h and returning to baseline at 24 h compared with that of control group (n = 14) (Fig. 2B). After CSD, the periorbital mechanical thresholds of the mice were significantly reduced, showing obvious mechanical hyperalgesia.

Compared with those of control group (n = 8), the time of facial grooming and freezing behaviors in the CSD group (n = 8) were significantly longer (*p* = 0.0228 and *p* = 0.0024, respectively) (Fig. 2C, D). However, the time of body grooming and head shaking were not significantly different between the CSD and control group. It is worth noting that there was no significant difference in travel distance in the central area between the CSD and control groups, which indicated that the mice did not produce anxiety-like behaviour after single CSD stimulation (Fig. 2E).

There were no differences in the time spent, travel distance and entries into the open arms between the CSD group (n = 8) and the control group (n = 6), indicating that single CSD did not produce obvious anxiety-like behaviours (Fig. 2G–I).

The light-aversion was more obvious in CSD group (n = 31) than in the control group (n = 27). Each mouse was tracked by the infrared array for 20 min divided into four 5-min intervals. Optogenetic induction of CSD resulted in significant light-aversive behaviour. The total distance decreased in the CSD group at 5, 10 and 15 min (*p* = 0.036, *p* = 0.001, and *p* < 0.001 respectively), as evidenced by a significant reduction in total distance (*p* < 0.001) (Fig. 2K, L). Since the total distance between control and CSD mice showed significant differences, we used the percentage of distance in the light box for comparison. The overall percentage of distance in light was significantly decreased in the CSD group, with a significant overall effect (*p* = 0.024) (Fig. 2M). Mice in the CSD group traveled less in the light box at 5, 10 and 15 min intervals (*p* = 0.015, *p* = 0.0099, and *p* = 0.048, respectively) (Fig. 2N). In addition, the mice in the CSD group rested more than those in the control group at 5, 10, 15 and 20 min in the dark box (*p* < 0.001, *p* < 0.001, *p* = 0.003 and *p* = 0.025, respectively) and the total rest time was more in CSD group (*p* < 0.001) (Fig. 2O, P).

### Mapping connectivity subnetworks of mouse brain activation after CSD

**Fig. 3:**
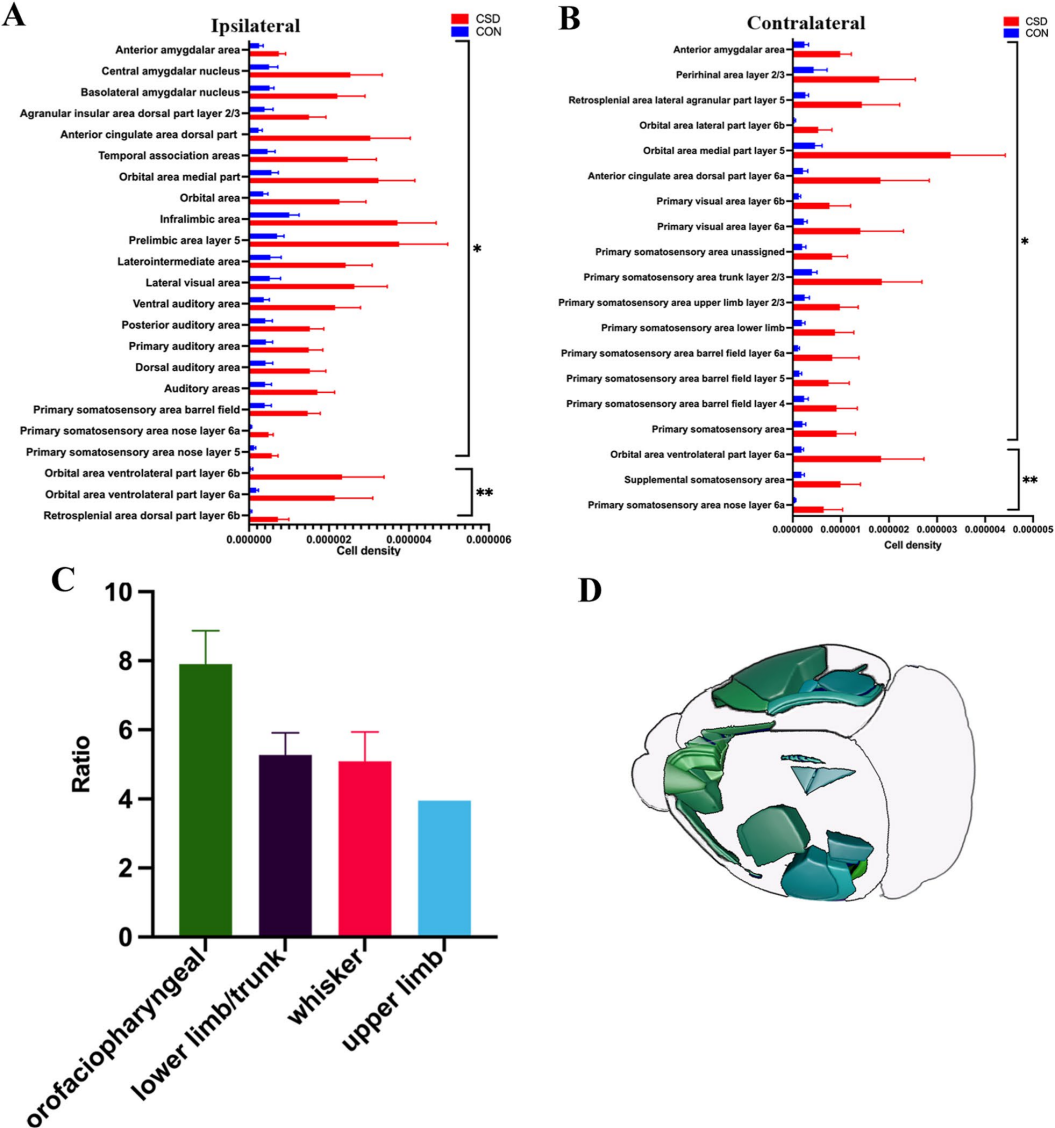
C-fos activation in mouse brains after CSD. **A**, **B** Automated segmentation of the cell density by anatomical regions sorted by *p* values. Data were represented as mean ± SEM, *p < 0.05, **p < 0.01, ***p < 0.001. **A** The regions in ipsilateral hemisphere was shown. **B** The regions in contralateral hemisphere was shown. **C** The ratio of orofaciopharyngeal, lower limb/trunk, whisker and upper limb subnetwork were shown. **D** The activated brain areas were not contiguous

Systematic mapping of brain activity provides the opportunity for an unbiased investigation of the activated regions after CSD. Mice were given visual cortex stereotaxic injections of photosensitive protein virus or control virus, followed by optogenetic stimulation and their brains were then processed (n = 5 for the CSD group and control group, respectively). Mapping brain activity via automated analysis of c-fos was a high throughput and accurate method and could greatly reduce the bias of manual counting. Generally, n = 3 in each group was considered to be sufficient for c-fos counting [14]. Therefore, n = 5 was a sufficient sample size [24]. Because the brain areas would have the different volumes in each mouse, we chose the c-fos density as a comparison standard. After comparing the c-fos density of the CSD group with that of the control group, VISoR detected significant activity increments in some regions after CSD (Fig. 3A, B).

**Fig. 4:**
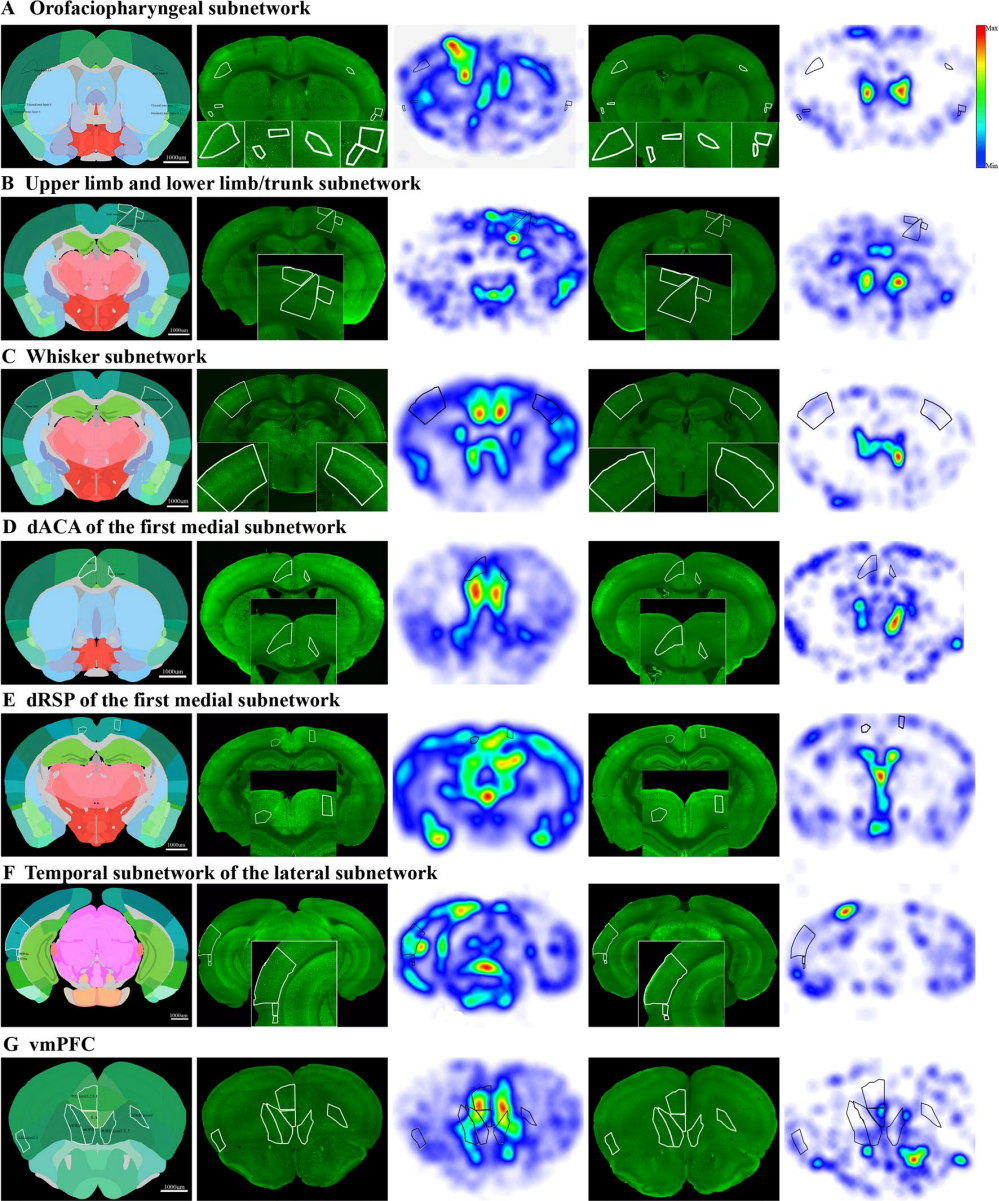
Increased neuronal activity of connectivity subnetworks after CSD. Automated analysis of c-fos positive cell density in mouse brains harvested 3 h after CSD (n = 5) or EYFP group (n = 5). The raw data and c-fos density heat map were 25-um coronal section. **A** Active areas in orofaciopharyngeal subnetwork included primary somatosensory area nose layer 5 and 6, visceral area layer 6 and gustatory areas layer 4 on the ipsilateral hemisphere, and primary somatosensory area nose layer 6, visceral area layer 2/3 and 4 and gustatory areas layer 4 on the contralateral hemisphere. **B** The activation regions in upper limb subnetwork was primary somatosensory area upper limb layer 2/3 on the contralateral hemisphere. The contralateral primary somatosensory area lower limb and trunk layer 1 and 2/3 of lower limb/trunk subnetwork were activated. **C** Active areas in whisker subnetwork included ipsilateral primary somatosensory area barrel field and contralateral primary somatosensory area barrel field layer 4,5 and 6. **D** The activation regions in first medial subnetwork included ipsilateral anterior cingulate area dorsal parts (dACA) and contralateral anterior cingulate area dorsal part layer 6. **E** The activation regions in first medial subnetwork included ipsilateral retrosplenial area dorsal part (dRSP) layer 6 and contralateral RSP lateral agranular part layer 2/3 and 5. **F** Active areas in temporal subnetwork of the lateral subnetwork were temporal association areas (TEa), perirhinal area layer 6 and entorhinal area lateral part layer 6 on the ipsilateral hemisphere. **G** The activated regions in ventromedial prefrontal cortex (vmPFC) included ipsilateral orbital area medial part (mORB), orbital area ventrolateral part (vlORB), prelimbic area (PrL) layer 1, 2/3 and 5, infralimbic area (ILA), agranular insular area dorsal part (AId) layer 2/3 and contralateral mORB layer 2/3 and 5 and orbital area lateral part (lORB) layer 6

To visually parse the data, we divided the cortical regions according to the neural networks of the mouse neocortex [25]. Only the parts of somatosensory areas were activated in the four somatic sensorimotor subnetworks, including the orofaciopharyngeal subnetwork, upper limb subnetwork, lower limb/trunk subnetwork and whisker subnetwork (Fig. 4A–C). The average ratio of c-fos densities in the orofaciopharyngeal subnetwork (7.9) between the CSD and control groups was higher than that of the other sensorimotor subnetworks (upper limb subnetwork: 3.9, lower limb/trunk subnetwork: 5.3, whisker subnetwork:5.1) (Fig. 3C), which confirmed that the orofaciopharyngeal subnetwork was preferentially activated to a higher degree, suggesting that it played a more important role after CSD.

The activated areas of the first medial subnetwork were the retrosplenial area and anterior cingulate area of dorsal parts (Fig. 4D, E). The activated temporal subnetwork of the lateral subnetwork included the temporal association area (TEa), perirhinal area layers 2/3 and 6, and lateral entorhinal part layer 6 (Fig. 4F). In addition, the prelimbic area, infralimbic area, anterior cingulate area dorsal part and orbital area medial part, which make up the medial prefrontal cortex (mPFC), were activated after CSD. Additionally, the active orbitofrontal area includes the ventrolateral orbital area (vlORB) and lateral orbital area (lORB). The ventromedial PFC composes mPFC, vlORB, lORB and agranular insular area, which were activated after CSD (Fig. 4G).

Interestingly, the activated brain areas were not contiguous: the activations were all sensory-related brain areas (Fig. 3D). Therefore, the regions of pain and pain-related emotional processes were activated; however, they were dispersed in the cortex. These results demonstrated that CSD originating from the visual cortex could selectively activate part of pian-related regions, suggesting that CSD was a complex sensory physiological phenomena.

### Mapping functional networks of CSD induced brain activity

**Fig. 5:**
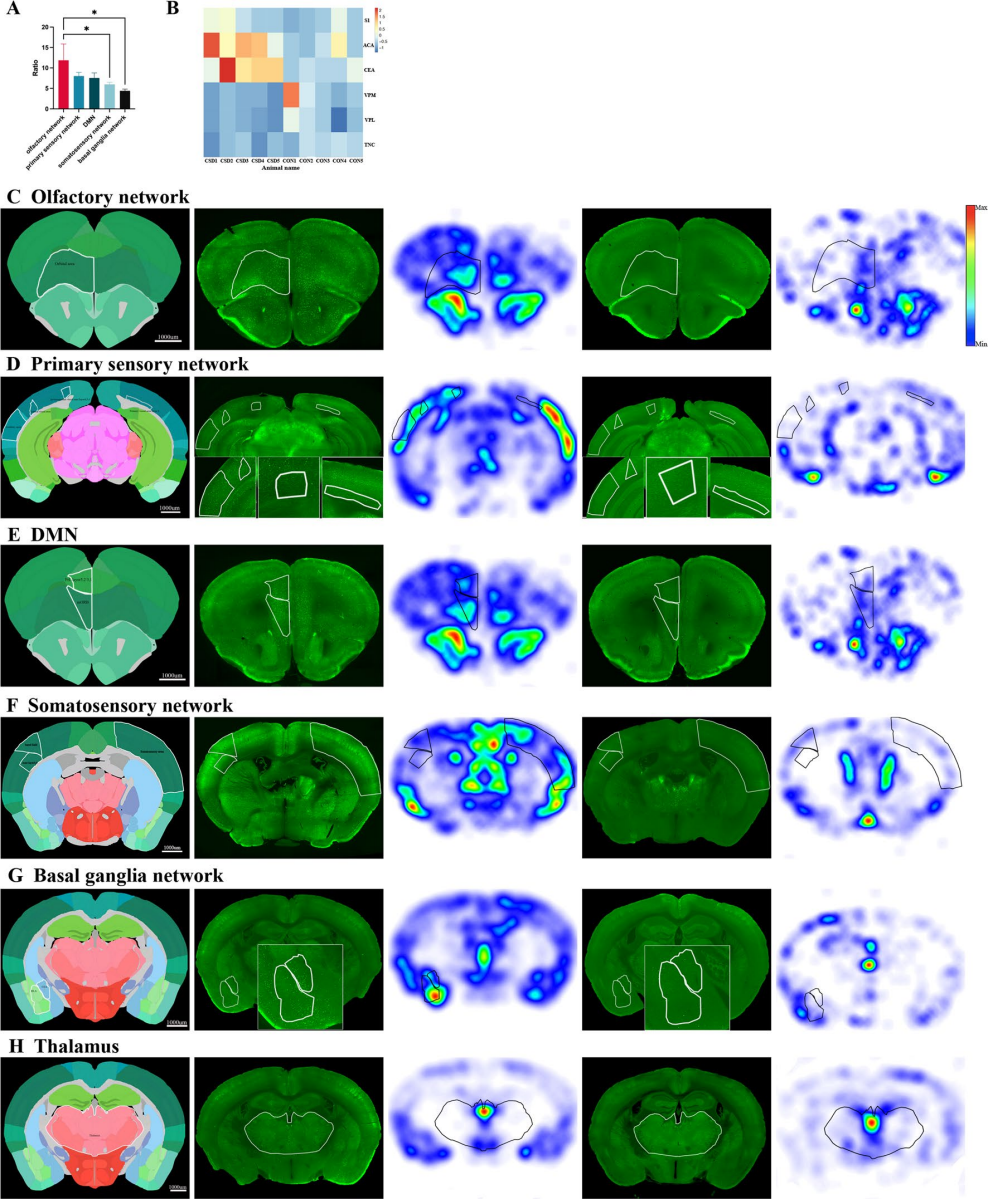
Increased neuronal activity of functional networks after CSD. **A** The activation level of olfactory network was significantly greater than somatosensory network than basal ganglia network (*p* = 0.049 and *p* = 0.035). Data were represented as mean ± SEM, *p < 0.05. **B** Z-score heatmap of 6 brain regions from 10 mice, indicating that ventral posteromedial nucleus of the thalamus(VPM), ventral posterolateral nucleus of the thalamus(VPL) and trigeminal nucleus caudalis(TNC) were not activated, however primary somatosensory areas (S1), anterior cingulate area dorsal parts (dACA) and central amygdalar nucleus (CEA) were significantly activated in CSD group compared with control group. **C**–**H** Automated analysis of c-fos positive cell density in mouse brains harvested 3 h after CSD (n = 5) or EYFP group (n = 5). The raw data and c-fos density heat map were 25-um coronal section. **C** The activated area of olfactory network was ipsilateral orbital area. **D** Active areas in primary network included ipsilateral auditory area, lateral visual area and posteromedial visual area layers 4, 5 and 6, and contralateral primary visual area layer 6. **E** The activation regions in default mode network (DMN) were orbital area medial part (mORB) and prelimbic area (PrL) layers 1,2/3 and 5. **F** The increased activity areas in somatosensory network included ipsilateral primary somatosensory area nose layers 5 and 6 and primary somatosensory area barrel field, and contralateral somatosensory area. **G** The ipsilateral amygdala was the major activation nuclei in basal ganglia network. **H** The thalamus was not activated compared with EYFP group

According to the functional network [26], we observed that the activation level of the olfactory network was greater than that of the primary sensory network (motor-visual-auditory areas), the default mode network (DMN), the somatosensory network and the basal ganglia network with the ratio(the ratio of the region c-fos density of the CSD group compared with the control group) representing the degree of activation of brain regions (Fig. 5A). Surprisingly, neither the thalamus nor the TNC was activated, indicating that CSD might induce a central disorder and peripheral input might be not required (Fig. 5B).

The degree of olfactory network activation was the highest, which might be related to the highly developed rhinencephalon in mice. The activated area of the olfactory network was the orbital area (Fig. 5C). Further, the activated areas of the primary sensory network included the auditory and visual areas (Fig. 5D). Visual-related brain regions were all activated bilaterally. Moreover, the orbital area medial part and prelimbic area of DMN showed increased activity (Fig. 5E). The increased activity of the ipsilateral primary somatosensory area nose, primary somatosensory area barrel field and contralateral somatosensory area were part of the somatosensory network (Fig. 4F). The major activation nucleus in the basal ganglia network was the amygdala, which was an important sensory integration nuclei (Fig. 5G). However, the thalamus was not activated, which is the relay of the periphery sensory (Fig. 5H).

### Immunofluorescence results

**Fig. 6:**
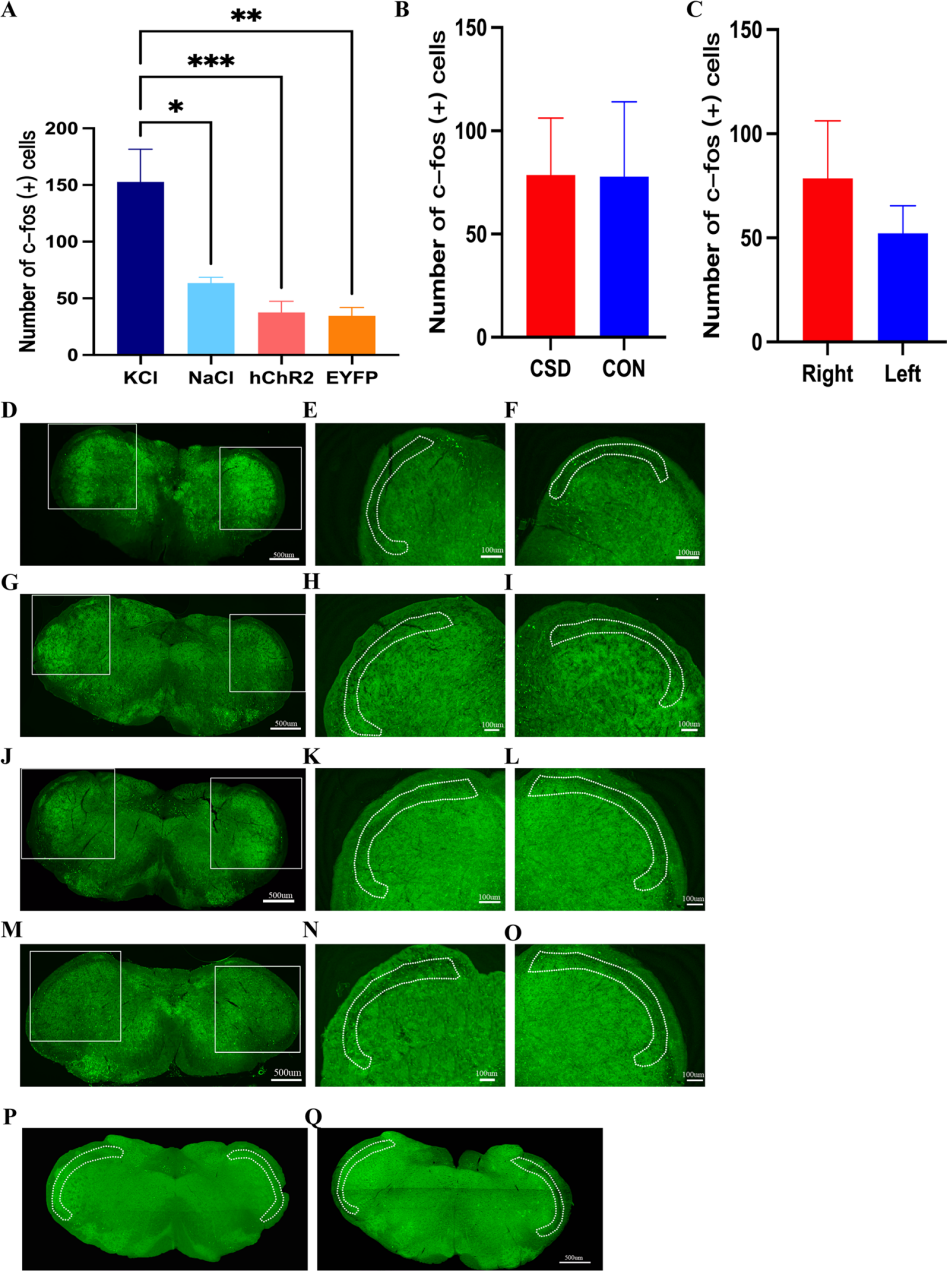
The expression of c-fos in I and II laminae of TNC after CSD. The dotted frame represents the I and II laminae of TNC. **A** In KCl model, CSD resulted in significant c-fos expression in ipsilateral I and II laminae of TNC compared with NaCl group (*p* = 0.0168, t-test), optogenetic CSD group (hChR2 group) (*p* = 0.001, t-test) and EYFP group (*p* = 0.0012, t-test). **B** The I and II laminae of TNC in VISoR images, the ipsilateral c-fos expression was not significant between CSD and control group. **C** In CSD group, the c-fos in ipsilateral and contralateral was not significant. **D** Images of c-fos expression in I and II laminae of TNC in KCl group. **E** The contralateral of TNC in KCl group. **F** The ipsilateral of TNC in KCl group. **G** Images of c-fos expression in I and II laminae of TNC in NaCl group. **H** The contralateral of TNC in NaCl group. **I** The ipsilateral of TNC in NaCl group. **J** Images of c-fos expression in I and II laminae of TNC in optogenetic CSD group. **K** The contralateral of TNC in optogenetic CSD group. **L** The ipsilateral of TNC in optogenetic CSD group. **M** Images of c-fos expression in I and II laminae of TNC in control group. (**N**) The contralateral of TNC in control group. **O** The ipsilateral of TNC in control group. **P** VISoR images of c-fos expression in I and II laminae of TNC in optogenetic CSD group. **Q** VISoR images of c-fos expression in I and II laminae of TNC in control group

**Fig. 7:**
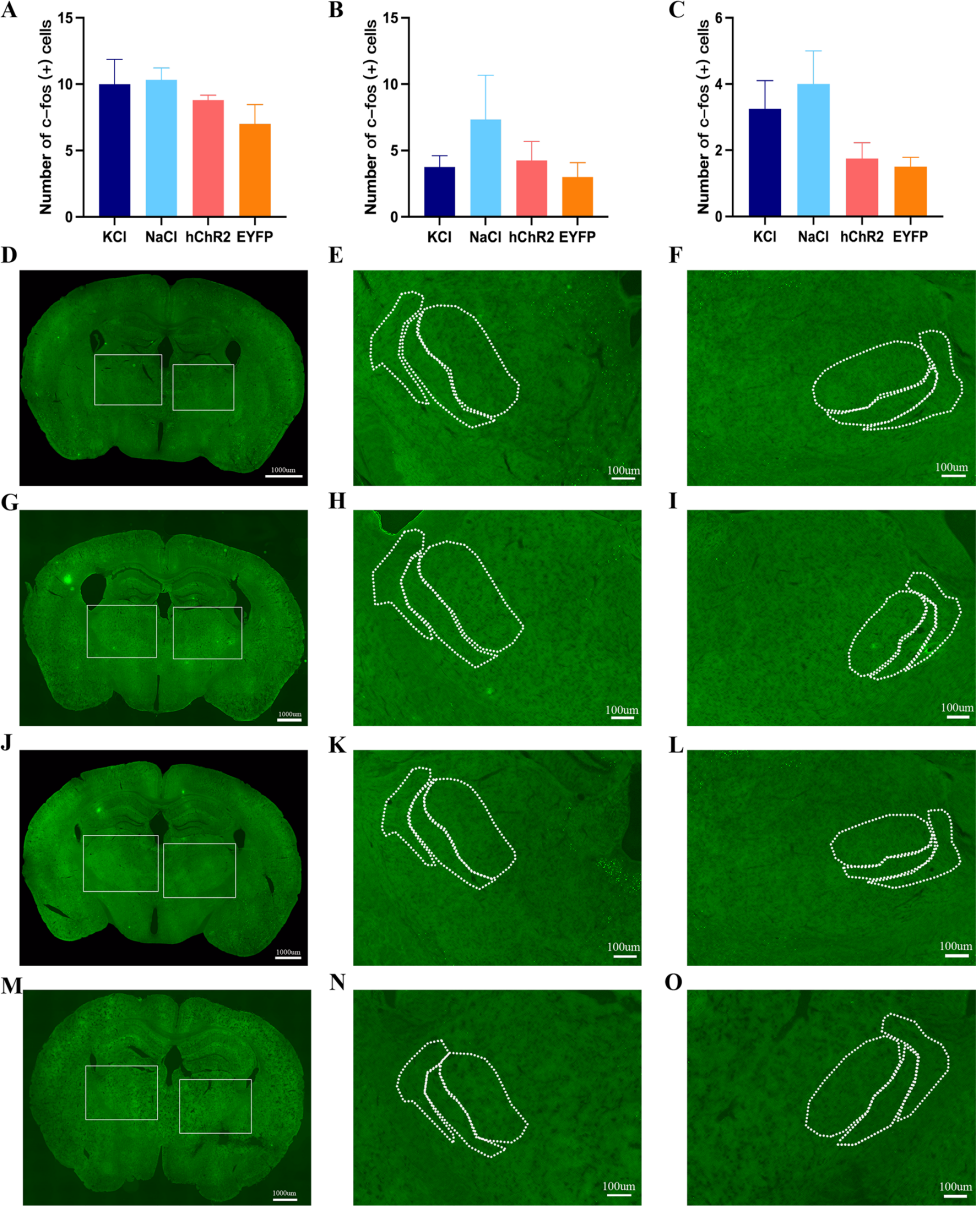
The c-fos expression in VPM, VPL and reticular thalamic nucleus (Rt). **A** The c-fos expression in ipsilateral VPM was not significant difference between KCl group, NaCl group, optogenetic CSD group and EYFP group. **B** The c-fos expression in ipsilateral VPL was not significant difference between KCl group, NaCl group, optogenetic CSD group and EYFP group. **C** The c-fos expression in ipsilateral Rt was not significant difference between KCl group, NaCl group, optogenetic CSD group and EYFP group. **D** Images of c-fos expression in thalamus in KCl group. **E** The contralateral of VPM, VPL and Rt in KCl group. **F** The ipsilateral of VPM, VPL and Rt in NaCl group. **G** Images of c-fos expression in thalamus in NaCl group. **H** The contralateral of VPM, VPL and Rt in NaCl group. **I** The ipsilateral of VPM, VPL and Rt in NaCl group. **J** Images of c-fos expression in thalamus in optogenetic CSD group. **K** The contralateral of VPM, VPL and Rt in optogenetic CSD group. **L** The ipsilateral of VPM, VPL and Rt in optogenetic CSD group. **M** Images of c-fos expression in thalamus in EYFP group. **N** The contralateral of VPM, VPL and Rt in EYFP group. **O** The ipsilateral of VPM, VPL and Rt in EYFP group

IN the KCl model, CSD resulted in significant c-fos expression in ipsilateral I and II laminae of TNC compared with the NaCl group (*p* = 0.0168), optogenetic CSD group (*p* = 0.001) and EYFP group (*p* = 0.0012) (Fig. 6A and D–O). After manually counting the I and II laminae of TNC in VISoR images, we found that the ipsilateral c-fos expression was not significantly different between the CSD and control group. In the CSD group, the c-fos expressions in the ipsilateral and contralateral side was not significantly different (Fig. 6B, C and P, Q). The c-fos expressions in ipsilateral and contralateral VPM, VPL and Rt was not significantly different among the KCl group, NaCl group, optogenetic CSD group and EYFP group (Fig. 7A–O).

## Discussion

In this study, we established an improved CSD model without irritating the dura, which could well mimic the behaviour of headache and photophobia. Additionally, an unbiased, broad pipeline using c-fos as a proxy for cell activity with high throughput was used to depict the neuronal networks activating after CSD. CSD is associated with the activation of multiple networks, including the olfactory network, primary sensory network, DMN, somatosensory network and basal ganglia network. Both the VISoR and immunofluorescence staining method showed that the TNC and thalamus were not activated in the optogenetic CSD model.

### Optogenetic CSD model could induce headache and photophobia

The traditional methods for inducting CSD are KCl microinjection, pinprick and electrical stimulation, which inevitably stimulate the dura, causing dural nociceptive afferents to activate TNC neurons, thus confusing the experimental results [27–29]. Although the optogenetic stimulation in Thy-1 mice does not injure the dura, CSD is rarely induced in the occipital cortex because of the lower ChR2 expression in occipital cortex in Thy-1 mice [8]. Our optogenetic approach does not cause cortical injury or irritation to the dura mater, and it activates the glutamatergic neurons at a lower light threshold in any cortical region.

Facial grooming is defined as a spontaneous expression of aversion caused by facial pain. Earlier studies showed that noxious mechanical stimulation of the faces of rat induced facial grooming [30, 31]. Although it is impossible to measure headache in mice, the facial grooming is considered as a sign of facial pain in freely moving rodents [32]. The behavioural results showed that the time of facial grooming in the CSD group was significantly longer than that in the control group, so we could infer that headache occurred in mice after CSD. Cutaneous allodynia of the periorbital region is well accepted as another symptom of headache [33]. We observed that CSD could trigger the allodynia of the periorbital region at 30 min and resolve at 24 h, respectively. Additionally, we found that the somatosensory network was activated after CSD, which could be related to the headache. Particularly, the orofaciopharyngeal subnetwork was preferentially activated to a higher degree, suggesting that the larger changes to the sensation of the head and face. Previous studies have found that CSD induces sharpening of the central sensory region and suppression of the surrounding region in rats [34]. The sensory map changes cause a sustained attenuation of the normal decremental response of the sensory cortex to repetitive stimulation, leading to decreased adaptation to stimulation and sustained sensation amplification [34]. The sensory amplification is associated with hyperexcitability and disinhibition of sensory network with increased release of cortico-cortical glutamatergic neurons and disinhibition of interneurons [35, 36]. Therefore, CSD-induced somatosensory network activation and changes in the sensory map might lead to an amplified sensory response, which induces headache.

Freezing behaviour is the most prominent behaviour in the traditional CSD model. The increased freezing time in the CSD group confirms the successful establishment of the optogenetic CSD model. Freezing is considered as a reflection of anxiety reaction [15]. However, open field and elevated plus-maze test demonstrated that single CSD could not induce anxious behaviours. In contrast, repeated CSD significantly increased the anxiety-like behaviors [37]. It is possible that structural and functional changes in cortico-limbic circuits, such as anterior cingulate cortex, involved in anxiety and depression may occur after repeated CSD attacks. Therefore, freezing is not the anxious behaviors in CSD mice. On the contrary, freezing might be a self-protective behaviour and expression of fear resulting from the headache. In addition, the amygdala was also activated, which was considered as an important emotion and sensory integration nuclei, and it was involved in the expression of freezing [22]. Headache would be aggravated by locomotion in migraineurs, and freezing could limit head and body movements in animals to relieve headache.

CSD also induced the obvious photophobia in mice. Clinically, over 90% of migraineurs report photophobia [38]. Visual symptoms could be the most prominent manifestations besides headache in migraineurs. Multiple studies have suggested that the photophobia is due to visual stimulation amplification and is associated with hyperexcitability of the visual network [35, 39]. The results of VISoR demonstrated that the primary sensory network was activated, including the visual cortex. In addition, studies have shown that the visual network of migraineurs is hyperexcitable and lacks normal habituation: (1) electroencephalogram and magnetoencephalography studies show that the visual network is hyper-responsive to visual stimuli [39, 40]; (2) fMRI study shows the increased visual cortex activation to light [41]. Therefore, photophobia is the result of hyperexcitation of the visual network.

The motility of the CSD group was reduced and the resting time in the dark zone was increased. It is speculated that the mice travel to avoid light until they arrive at the dark zone, where they rest. We surmise that movement and light aggravate headache, which may correspond to pain aggravation by routine activity and rest in dark in migraineurs.

### CSD generates multicircuit of neuronal activity

The propagation of CSD is continuous in the cortex. However, not all brain areas in which CSD propagated are activated. We infer that the discontinuous activation regions are based on the structural connectivity network, including the somatic sensorimotor, first medial and temporal subnetwork of the lateral subnetwork, and that there are synaptic connections between the activated brain regions [25]. It is well documented that CSD can induce the release of signaling molecules that cause cellular activation and vasodilation [42]. It is likely that the signal that CSD activates neurons is transmitted through synapses between neurons. Therefore, we deduce that the subnetworks activation occurs through the circuits between subnetworks.

### Optogenetic CSD could induce cortical pain

The VISoR and immunofluorescence staining methods showed that the TNC and thalamus were not activated in the optogenetic CSD model. Similar expression of c-fos in the TNC was found in the optogenetic CSD group, NaCl group and EYFP group, however the pain-related regions were significantly activated in CSD group, indicating that activation of glutamatergic neurons to induce CSD may cause headache by directly activating cortical pain-related regions rather than activating TNC by meningeal afferents. However, activation of the ipsilateral TNC was found in the KCl group compared with the optogenetic CSD group, NaCl group and EYFP group, respectively. There might be two possible mechanism that cause the difference. One is that the CSD pathway activated by KCl is different from that caused by the activation of glutamatergic neurons. Although it is generally believed that potassium ions and glutamate are important substances that induce CSD, the pathway of potassium- and glutamate-induced CSD is still unclear [43]. It might be that KCl-induced CSD could activate meningeal nociceptors and trigeminovascular pathway underlying the headache phase [2, 3], while activation of glutamatergic neurons to induce CSD may cause headache by directly activating cortical pain-related regions. And another explanation might be that it was KCl rather than CSD that activated the meningeal nociceptors. Fioravanti and Ingvardsen have found that there is a positive correlation between the number of c-fos positive cells in TNC and the number of KCl injections rather than the number of CSD [44, 45]. Additionally, the pinprick to the cortex could not increase c-fos expression in TNC, but hypertonic NaCl to dura could induce the expression of c-fos in TNC [44, 45]. Thus, the c-fos expression in TNC may result from the stimulation of KCl to dura rather than the CSD. Moreover, there is no study showing the activation of contralateral VPM in CSD model. Even in awake CSD animals, only the ipsilateral Rt is activated and Fu et al. found that ipsilateral VPM is activated in awake animals, which is more like a direct subcortical spreading of the CSD, and the Rt and VPM will not be activated if the experiment is conducted under anaesthesia [20, 46]. CSD was induced under anaesthesia in all mice used for c-fos counting and behavioural analysis, and the mice still showed the behaviours related to headache and photophobia, which demonstrates that cortical activation could cause headache without activation of the thalamus and TNC.

CSD originating from the occipital lobe could cause cortical pain, which was manifested as headache and photophobia, similar to migraine symptoms, and may serve as a new model of typical migraine with aura. Electrical stimulation (ES) of dural mater surrounding superior sagittal sinus could well mimic the process and behavior of migraine. Previous studies have found that ES could suppress the outward K^+^ current and increase the excitability of neurons in TG, which induces headache [47]. Therefore the ES model is a relatively ideal model for migraine without aura. However, ES model could not represent the migraine with aura and the optogenetic CSD model can better mimic the pathogenesis and process of migraine with aura. And there might exist different migraine pathogenesis in optogenetic CSD. By comparing the different activated brain networks in the ES model and CSD model could elaborate on different mechanisms underlying migraine.

CSD originating from the occipital lobe is closely related to the occurrence of headache. It is reported that the occipital lobe not only plays an important role in the pathogenesis of migraine, but is also closely related to headache after ischemic stroke and post-ictal headache [38, 48–50].

A recent meta-analysis showed that the most typical imaging change in migraine patients with and without aura is structural atrophy and functional hyperactivity of the V3 and V3A, suggesting that the occipital lobe plays an important role in the pathogenesis of migraine [38]. Moreover, a previous fMRI revealed that CSD originated from the V3/V3A, which may be due to the hyperactivity of the V3/V3A leading to the occurrence of CSD and headache [51].

It has been reported that there is an almost two-fold prevalence of headache after ischemic stroke in the posterior circulation stroke than that in anterior circulation stroke, and migraine-like headaches accounts for more than half of the headaches after stroke in the posterior circulation [48]. The regions in the posterior circulation, such as the occipital lobe, may play an important role in the mechanism of headache development. Headache after ischemic stroke is considered to be associated with cortical as opposed to subcortical or deep infarcts [48]. A previous study found that headache was related to infarcts involving the insular and somatosensory cortex, suggesting that structural and functional abnormalities of the cortex can contribute to headaches [52].

Post-ictal headache occurs in 45% of epilepsy patients, of which 50% are migraine-like headaches [49]. The incidence of post-ictal headache is significantly related to the location of epilepsy. Many studies have found that the post-ictal headache is more common in those with occipital epilepsy than in those with epilepsy originating in the frontal or temporal lobes, and most post-ictal headache is migraine-like headaches in patients with occipital epilepsy, indicating that the occipital lobe is strongly associated with migraine-like headaches [49, 50]. Migraine with aura is significantly more frequent in patients with comorbidity of migraine and epilepsy, which supports the hypothesis that CSD is the common pathophysiology of migraine and epilepsy [53]. A meta-analysis speculated that the possible mechanism of epileptic headache might be the activation of pain-causing brain regions, so we speculate that CSD could activate cortical pain-causing brain regions to induce headache [49].

Overall, abnormalities of the occipital lobe can cause headache in various diseases and the common mechanism of headache might be the CSD originating from the occipital lobe, which could directly activate cortical pain-related brain regions, such as somatosensory network and primary sensory network, without activation of the TNC to induce cortical pain.

### Caveats and limitations

There are several caveats in our study. First, c-fos has low temporal resolution. Its expression usually lags neuronal activity by 30 min because c-fos relies on the translational process. Second, the c-fos-based network should not be simply interpreted as a complete brain activity map because c-fos is a member of the IEGs family and neuronal activities may induce other IEGs. Third, the colocalizations are not performed. We can not differentiate the activated cells to clear whether the activated neuron or glial, or which type of neuron.

In conclusion, we established an improved CSD model using optogenetic technique that could mimic the behaviours of headache and photophobia. Furthermore, we found that the CSD activates multiple sensory-related networks and generates multicircuit patterns of c-fos-tagged neuronal activity. Lastly, we found that the optogenetic CSD could activate part of central sensory regions without activating of the thalamus and TNC to induce cortical pain.

## Data Availability

The data that support the findings of this study are available from the corresponding author on reasonable request.

## Abbreviations

CSD: Cortical spreading depression
KCl: Potassium chloride
TNC: Trigeminal nucleus caudalis
IEGs: Immediate early genes
VISoR: Volumetric imaging with synchronized on-the-fly-scan and readout
CamkIIa: Calcium/calmodulin-dependent protein kinase II alpha
PBS: Phosphate-buffered saline
VPM: Ventral posteromedial thalamic nucleus
VPL: Ventral posterolateral thalamic nucleus
Rt: Reticular thalamic nucleus
vlORB: Ventrolateral orbital area
Tea: Temporal association area
mPFC: Medial prefrontal cortex
lORB: Lateral orbital area
DMN: Default mode network
fMRI: Functional magnetic resonance imaging
CGRP: Calcitonin gene-related peptide
ES: Electrical stimulation
